# Three solid malignancies and a myelodysplastic syndrome with a protracted course after kidney transplantation

**DOI:** 10.1093/ckj/sft042

**Published:** 2013-06

**Authors:** Friedrich C. Prischl, Sonja Burgstaller, Manfred Wallner, Eva Seiringer, Patrick Dinkhauser, Walter Pauer, Josef Thaler

**Affiliations:** 1 4th Department of Internal Medicine (Nephrology and Haematology/Oncology), Klinikum Wels-Grieskirchen, Wels, Austria; 2 Department of Urology, Klinikum Wels-Grieskirchen, Wels, Austria

**Keywords:** breast cancer, kidney transplantation, leukaemia, malignancy, transitional cell carcinoma

## Abstract

Although a well-known complication after transplantation, multiple non-skin malignancies within a patient are rare. We report on a kidney transplant recipient who over the course of 20 years developed breast cancer twice, a uroepithelial carcinoma, and myelodysplasia transforming into acute leukaemia. Breast cancer was treated as usual. The transitional cell carcinoma was managed with partial cyst ureterectomy with transposition of the native ureter to the graft. Withdrawal of immunosuppression followed under a “watchful waiting” regime. In conclusion, alertness is requested regarding development of malignancies. Creative solutions are necessary in the management of such patients. Under exceptional circumstances, withdrawal of immunosuppression may be an option.

## Introduction

The occurrence of malignancy after organ transplantation is well known. Large registries show a 3- to 5-fold increased risk compared with age-matched controls [1]. The appearance of certain entities varies widely, with lip and skin cancer yielding the highest standardized incidence rates (SIR) and others, including breast cancer, with even lower SIR among transplant recipients compared with the general population [2]. Risk factors for non-skin malignancy after kidney transplantation are higher age, male sex and white and non-Hispanic ethnicity [3]. The cumulative cancer incidence increases with time [4, 5]. Little is known, however, about multiple non-skin malignancies within an individual transplant recipient. We report on a patient with an >20-year lasting course after kidney transplantation who experienced two distinct breast cancers and a transitional cell carcinoma of the urinary tract and, despite withdrawing immunosuppression, developed myelodysplastic syndrome 19 months later transforming into acute terminal leukaemia.

## Case report

In February 1992, a 48-year-old woman with end-stage renal disease from analgesic nephropathy successfully underwent kidney transplantation. Immunosuppression consisted of cyclosporine and prednisone, the latter tapered and withdrawn after 6 years. Graft function was excellent for the next 10 years with serum creatinine values of 1.0–1.5 mg/dL, i.e. an estimated glomerular filtration rate (eGFR, according to MDRD formula) of 54–34 mL/min. In May 2002, left-sided breast cancer was detected (grade 2, pT2, N1bIII, N0, oestrogen receptor negative, progesterone receptor negative). The tumour was operated, followed by standard adjuvant chemotherapy, i.e. four cycles of cyclophosphamide 500 mg/m^2^, doxorubicine 50 mg/m^2^, 5-fluouracil 500 mg/m^2^ i.v., then 50 Gy cumulative dose radiotherapy of left breast and 66 Gy cumulative boost radiation of the resected tumour area and finally four cycles of docetaxel 75 mg/m^2^. Regular follow-up excluded recurrence of disease until August 2009 when another left-sided breast tumour was detected. Histological work-up after mastectomy showed a grade 2 (pT1a, NX), oestrogen (+++) and progesterone receptor (+++) positive and Her2neu negative (Her2neu-[IH]) adenocarcinoma, indicating the presence of a distinct second breast cancer. Life-long aromatase-inhibitor therapy with anastrazole 1 mg daily was initiated.

Shortly thereafter, acute transplant failure occurred with hydronephrosis resulting from ureteral stenosis. Placement of a ureteral catheter immediately reversed kidney failure and eGFR improved from 10 to 30–35 mL/min. Repeated cytological examinations of urine were without pathology. Postrenal transplant failure occurred again because of obstruction of the ureteral catheter and was resolved by catheter replacement. Serological tests for polyoma virus infection were negative and urine cytology excluded decoy cells. In February 2010, ureteral catheter replacement failed because of a ureteral stenosis due to transitional cell carcinoma. The standard procedure would have been nephrectomy, bladder resection, radiation and/or chemotherapy depending on histological grading. However, this would have resulted in the need for dialysis with no guarantee of recovery.

Extensive discussions with the patient and her family followed. Radical surgery would have deteriorated the quality of life of our patient markedly as this was defined by freedom from dialysis as stated repeatedly by her. Therefore, it was decided to try tumourectomy with resection of the transplanted ureter and transposition of the patient's native ureter from the right kidney to the graft, followed by adjuvant chemotherapy. The patient agreed and successfully underwent surgery. As our female patient had a male organ donor, it could be ruled out that the origin of the cancer was the donor using fluorescence *in situ* hybridization analysis that excluded Y-chromosome-bearing cells. Histological work-up revealed high-grade transitional cell carcinoma and lymphangiosis but all cancer appeared to have been resected.

Whenever cancer was detected, management of immunosuppressive therapy was discussed. As repeated literature reviews in 2002, 2009 and in February 2010 showed no decreased cancer incidence of certain immunosuppressive regimes compared with others, cyclosporine was continued at doses to achieve whole-blood trough levels of 80–120 ng/mL until 2009 and unusually low levels (50–80 ng/mL) thereafter (determined by antibody-conjugated magnetic immunoassay, Abbott Architecture™ from 2008 onwards).

In April 2010, adjuvant chemotherapy with gemcitabine (1000 mg/m^2^, reduced to 750 mg/m^2^ in cycles 3 and 4) and carboplatin (dose calculated according to AUC 5, taking into account eGFR) was initiated. After the fourth cycle, chemotherapy was stopped because of grade 4 toxicity (urosepticaemia, candidaemia, *Clostridium difficile* enteritis). Doses of immunosuppression were always reduced during chemotherapy and were eventually stopped in times of septicaemia. In August 2010, prolonged and life-threatening septicaemia required us to stop administration of immunosuppressive drugs. As this worked well with regard to graft function, after recovery we decided not to reinitiate immunosuppression and to follow a ‘watch-and-wait’ regime with frequent visits also considering the repeated development of malignancies under immunosuppression. Fortunately, the patient fully recovered, and her positive attitude was outstanding. eGFR values of 22–32 mL/min were indicative of immune tolerance and a still functioning kidney transplant.

In February 2012, progressive anaemia, leucokytopenia and thrombocytopenia led to bone marrow exploration showing secondary myelodysplastic syndrome (del 7q, IPSS intermediate-2 risk). Azacytidine (75 mg/m^2^ Day 1–7, repeated every 28 days) treatment was commenced due to risk classification and transfusion-dependent anaemia and thrombocytopenia. The patient showed a good response in the first 4 months. In September 2012, transformation into acute myeloid leukaemia was confirmed by bone marrow examination. The patient died 20 years and 7 months after kidney transplantation from leukaemia (serum creatinine 1.4 mg/dL, i.e. eGFR 37 mL/min).

## Discussion

The number of patients with long-term function of transplanted kidneys is rising steadily. This success results from improved handling of immunosuppressive therapy and patient management in general, but is reached at the price of accelerated occurrence of malignancies [1]. When severe infections and cardiovascular events are managed successfully, cancer is going to become a major cause of death. Data from the Australian and New Zealand Registry indicate non-skin cancer to affect 25% of long-term survivors after kidney transplantation, 70% of whom subsequently die from cancer [6]. Even in dialysis patients the risk of cancer has increased markedly [7]. According to textbooks, the management of transplant recipients consists of reduction of immunosuppression and surgery, radiotherapy and chemotherapy following the usual standards [8].

Our patient had no family history of cancer and none of the risk factors described [3] apart from analgesic nephropathy. Stewart *et al.* [9] analysed kidney and urinary tract cancer in dialysis patients and found an outstanding SIR for both in patients with analgesic nephropathy. Moreover, in kidney recipients followed for 30 years [10], urological cancer was the second most prevalent malignancy (15.6%) after skin tumours (44.4%).

Apart from skin cancer, rarely several tumours occur in an individual. Our patient had three distinct solid tumours 10, 17 and 18 years after transplantation. Breast cancer was treated according to standards. More complicated was the management of transitional cell carcinoma. Standard radical surgery would have necessitated dialysis. Stimulating discussions with the patient led to an unusual solution with regional tumourectomy that resulted in histological freedom of cancer and respected the patient's wish to remain free from dialysis. Chemotherapy was given but was withheld thereafter due to life-threatening infections. Immunosuppression was also stopped. In retrospect, the following 2 years without evidence of transitional cell carcinoma recurrence favoured our therapeutic decisions.

Withdrawal of immunosuppression and ‘watchful waiting’ is uncommon. There are many studies on steroid withdrawal or conversion to two-drug- or single-drug/low-dose regimens in virtually all kinds of transplanted organs but complete withdrawal has been performed in single cases only (e.g. liver transplantation [11]) and always driven by special circumstances (e.g. renal transplantation [12]). In our patient, stopping immunosuppression was forced by life-threatening septicaemia and not reinitiating it thereafter was considered an option, also in the context of repeated malignancies developing even under unusual low doses of cyclosporine monotherapy. Watchful waiting worked well for 25 months and partly may have been responsible for the freedom from malignancy. No other routine monitoring of the graft function or rejection was done as frequent clinic visits and eGFR measurements showed stable graft function. Unfortunately, she then developed myelodysplastic syndrome with transformation into acute leukaemia. Most likely this was secondary to former chemotherapy for cancer [13]. In the current situation, no curative therapy was available and the patient deceased >20 years after transplantation with a still-functioning graft.

In conclusion, we learned from this case that high alertness is necessary with regard to development of malignancy, even late after transplantation. The unique course of our patient shows that creative adaptations of treatment standards have to be done to meet the patient's needs and to overcome even several distinct cancers. Withdrawing immunosuppression is an option in special circumstances, at least late in the course of disease. Finally, a patient's positive attitude may also be crucial.

## Conflict of interest statement

None declared.
